# Revisiting Solution-Based Processing of van der Waals Layered Materials for Electronics

**DOI:** 10.1021/acsmaterialsau.2c00034

**Published:** 2022-05-13

**Authors:** Jihyun Kim, Okin Song, Yun Seong Cho, Myeongjin Jung, Dongjoon Rhee, Joohoon Kang

**Affiliations:** † School of Advanced Materials Science and Engineering, Sungkyunkwan University (SKKU), Suwon 16419, Republic of Korea; ‡ KIST-SKKU Carbon-Neutral Research Center, Sungkyunkwan University (SKKU), Suwon 16419, Republic of Korea

**Keywords:** solution-based processing, van der Waals materials, 2D materials, liquid-phase exfoliation, electrochemical exfoliation, electronics

## Abstract

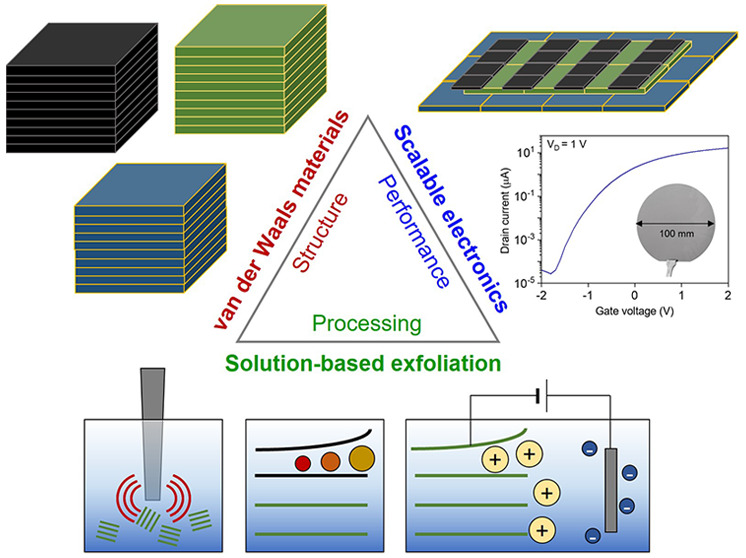

Following the significant discovery of van der Waals (vdW) layered materials with diverse electronic properties over more than a decade ago, the scalable production of high-quality vdW layered materials has become a critical goal to enable the transformation of fundamental studies into practical applications in electronics. To this end, solution-based processing has been proposed as a promising technique to yield vdW layered materials in large quantities. Moreover, the resulting dispersions are compatible with cost-effective device fabrication processes such as inkjet printing and roll-to-roll manufacturing. Despite these advantages, earlier works on solution-based processing methods (i.e., direct liquid-phase exfoliation or alkali-metal intercalation) have several challenges in achieving high-performance electronic devices, such as structural polydispersity in thickness and lateral size or undesired phase transformation. These challenges hinder the utilization of the solution-processed materials in the limited fields of electronics such as electrodes and conductors. In the meantime, the groundbreaking discovery of another solution-based approach, molecular intercalation-based electrochemical exfoliation, has shown significant potential for the use of vdW layered materials in scalable electronics owing to the nearly ideal structure of the exfoliated samples. The resulting materials are highly monodispersed, atomically thin, and reasonably large, enabling the preparation of electronically active thin-film networks via successful vdW interface formation. The formation of vdW interfaces is highly important for efficient plane-to-plane charge transport and mechanical stability under various deformations, which are essential to high-performance, flexible electronics. In this Perspective, we survey the latest developments in solution-based processing of vdW layered materials and their electronic applications while also describing the field’s future outlook in the context of its current challenges.

## 1 Introduction

Since the discovery of monolayer graphene isolated from bulk graphite by breaking weak van der Waals (vdW) interactions,^1−3^ post-graphene two-dimensional (2D) vdW layered materials have been extensively explored for more than a decade for use in electronic applications owing to their atomically thin structure and unique electronic properties.^4−11^ Various electronic classes of 2D vdW materials with structural analogy, such as metallic MXene,^12−14^ semimetallic graphene,^3,15,16^ semiconducting transition metal dichalcogenides (TMDCs) and black phosphorus,^4−7^ and insulating hexagonal boron nitride (h-BN) and montmorillonite,^3,8,11,17^ have been explored for use in electronic components (e.g., electrodes, channels, and dielectric layers). Furthermore, the synthesis of vdW heterostructures has been successfully demonstrated through the assembly of two or more vdW layered materials as building blocks with desired electronic properties.^3,18−23^ Most importantly, the atomically clean, dangling-bond-free surface of 2D samples enables the demonstration of numerous fundamental prototypes that rely on achieving efficient charge transport between the neighboring layers at vdW interfaces.^23−27^ Despite having prototyped high-performance electronics based on vdW heterostructures which push the intrinsic limits of such 2D materials, the complicated processes for vdW device fabrication (which include micromechanical cleavage-based monolayer sample preparations and microscopy-assisted precise transfer for heterostructure formations) hinder the scaling up of such devices while maintaining their performance for industrial-scale applications. To overcome these technological limitations, two main issues should be addressed: scalable production of high-quality vdW layered materials with high structural monodispersity and effective assembly of individual samples into vdW thin-film networks while minimizing device performance degradation.

Solution-based processing has been proposed as a promising route to address these challenges.^28−36^ Solution-based processing possesses almost unlimited scalability to produce an atomically thin 2D sample from nearly any existing layered bulk crystal. The resulting dispersions readily assemble into thin films on any substrate and at low temperatures. This compatibility with cost-effective, scalable, and low-temperature processes (such as inkjet printing and roll-to-roll manufacturing) is highly advantageous. Despite these promising aspects of demonstrated scalable processes, there remain several challenges including the generation of significant structural polydispersity (i.e., a broad distribution of thickness profiles), small lateral size, and chemical degradation.^7,33−35^ In this Perspective, we review the extent to which recent advances in solution-based processing of vdW layered materials for electronics have addressed such challenges. Please note that we will not cover other dimensional vdW materials including zero-dimensional colloidal quantum dots, fullerenes, and one-dimensional carbon nanotubes^20,37^ for in-depth discussion on vdW sheet-to-sheet contacts of 2D vdW materials and resulting properties.

## 2 Solution-Based Exfoliation of vdW Layered Crystals

**Figure 1:**
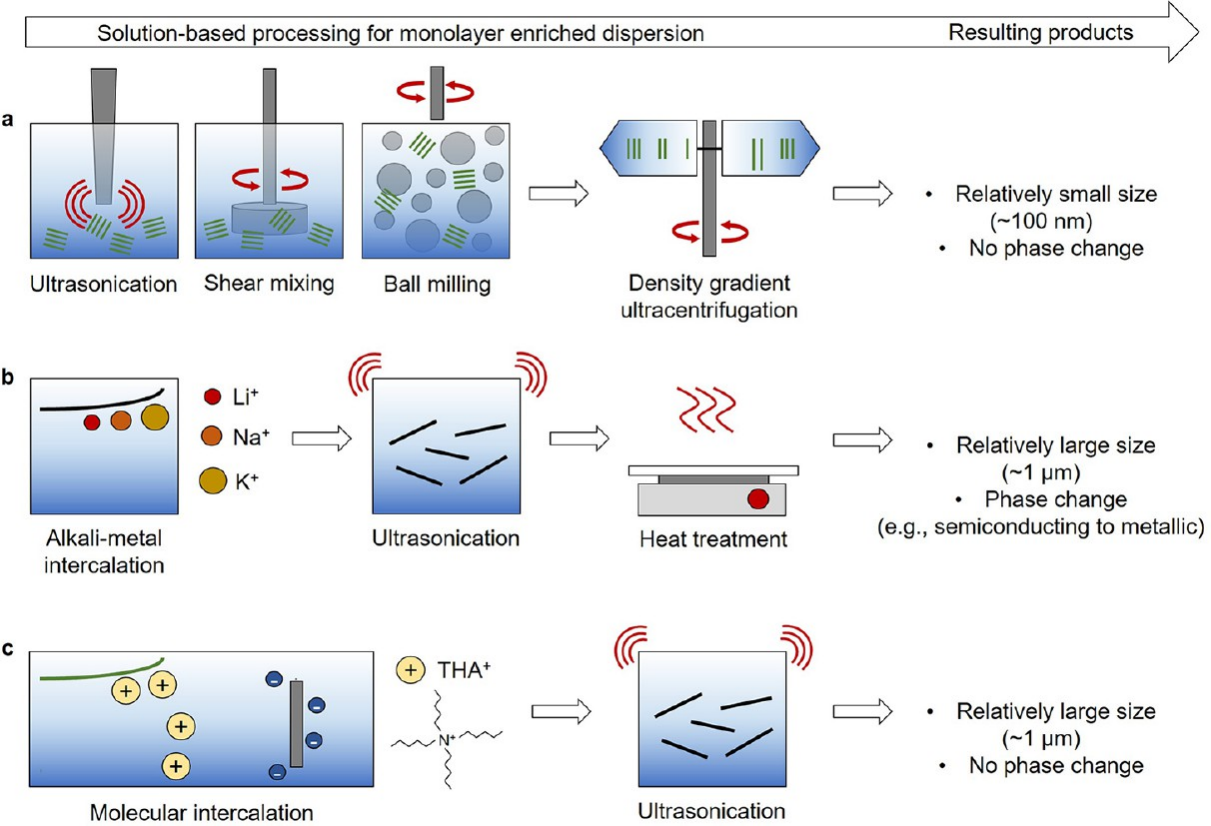
Schematics of solution-based processing of vdW layered materials. (a) Direct liquid-phase exfoliation of layered crystals and subsequent density gradient ultracentrifugation for precise structural sorting. (b) Alkali-metal intercalation-based exfoliation followed by heat treatment. (c) Molecular intercalation-based electrochemical exfoliation.

Solution-based production of vdW layered materials can be achieved using two primary approaches: direct liquid-phase exfoliation^8,30,33,34^ and intercalation-based van der Waals force weakening.^36,38−41^ Among solution-based approaches, we will not cover bottom-up methods such as liquid-phase precursor-assisted chemical vapor deposition,^42,43^ but we focus on the direct comparison of products generated from each top-down process, assuming that the starting material is identical to a piece of layered crystal. Figure 1a illustrates representative direct liquid-phase exfoliation approaches including ultrasonication, shear mixing, and ball milling. During this process, 2D vdW materials are exfoliated from their layered bulk crystals when the energy transferred is higher than the vdW interactions. For example, an applied energy higher than 2 eV per 1 nm^2^ is required to overcome vdW interactions to exfoliate graphene from layered graphite crystals.^44^ At the same time, the applied energy fragments bulk crystals, resulting in the relatively small lateral size of exfoliated nanosheets (∼100 nm). Because the lateral dimension of 2D nanosheets is a critical factor in designing electronic device structures (e.g., the channel length between source and drain), the lateral size loss during the process has been considered as a significant drawback of direct liquid-phase exfoliation methods. In addition, the resulting 2D samples possess polydispersity in the lateral dimension and thickness,^33,35,43^ which hinders efficient vdW sheet-to-sheet contact when forming thin-film network structures. The properties of 2D nanosheets are strongly dependent on their layer number due to strong quantum confinement; therefore, 2D polydispersity in thickness necessitates postprocessing to enrich the desired thickness of the samples; this is achieved through ultracentrifugation (i.e., density gradient ultracentrifugation).^8,33,45,46^ Density gradient ultracentrifugation was originally developed for the separation of biological macromolecules, and it is directly transferable to 2D nanosheet separation as their morphologies and buoyant densities are comparable to those of biological molecules.^33^ Depending on the desired structure of nanosheets, density gradient ultracentrifugation can be split into two categories: sedimentation-based density gradient ultracentrifugation and isopycnic density gradient ultracentrifugation.^33,34^ Generally, the former is utilized for lateral size sorting based on the size-dependent different sedimentation rates during ultracentrifugation, which is usually dominated by mass of the nanomaterial. Unlike the sedimentation-based process, the latter is dominated by buoyant density of the nanomaterial and thus suitable for thickness sorting. During ultracentrifugation, nanomaterials sediment to their corresponding isopycnic point in the density gradient medium. Following the isopycnic process, several bands are separated and visually observable in the tube, indicating nanomaterials are sorted based on their buoyant densities. The buoyant density of 2D nanosheets is strongly dependent on their thickness, and this approach enables highly monodisperse samples with different thickness. Although the postexfoliation process has enabled high 2D monodispersity in the required thickness, the resulting liquid-phase exfoliated samples possessing small lateral sizes continue to motivate alternative routes for efficient exfoliation. For example, alkali metal (e.g., Li, Na, and K) intercalation-based exfoliation approaches have been developed (as illustrated in Figure 1b) to produce dispersions with monolayer-enriched, relatively large MoS_2_ nanosheets. During the process, however, a thermodynamically stable trigonal phase of MoS_2_ (2H-MoS_2_) becomes a metastable octahedral phase (1T-MoS_2_), resulting in an undesired semiconducting to metallic phase transition due to the insertion of Li^+^ ions and associated electrons.^38^ To overcome this limitation, approaches for postprocessing have been proposed, which include mild annealing or laser exposure, to partially recover the semiconducting 2H-MoS_2_. However, the resulting high defect densities and the residual metallic phase hinder their full utilization for electronic applications.^38,47^ Alternatively, another intercalation-based exfoliation approach has been reported by Duan et al. to produce atomically thin MoS_2_ nanosheets without any phase transformations using quaternary alkyl ammonium molecules (i.e., tetraheptylammonium bromide, THAB) or other various ammonium molecules (e.g., (C_2_H_5_)_4_NBr, (C_3_H_7_)_4_NBr, (C_4_H_9_)_4_NBr, and (C_10_H_21_)_4_NBr) as an intercalant under electrochemical reaction (Figure 1c).^36^ The resulting atomically thin nanosheets possess relatively large lateral dimensions and, most importantly, retain their intrinsic semiconducting properties without any post-treatment.

**Figure 2:**
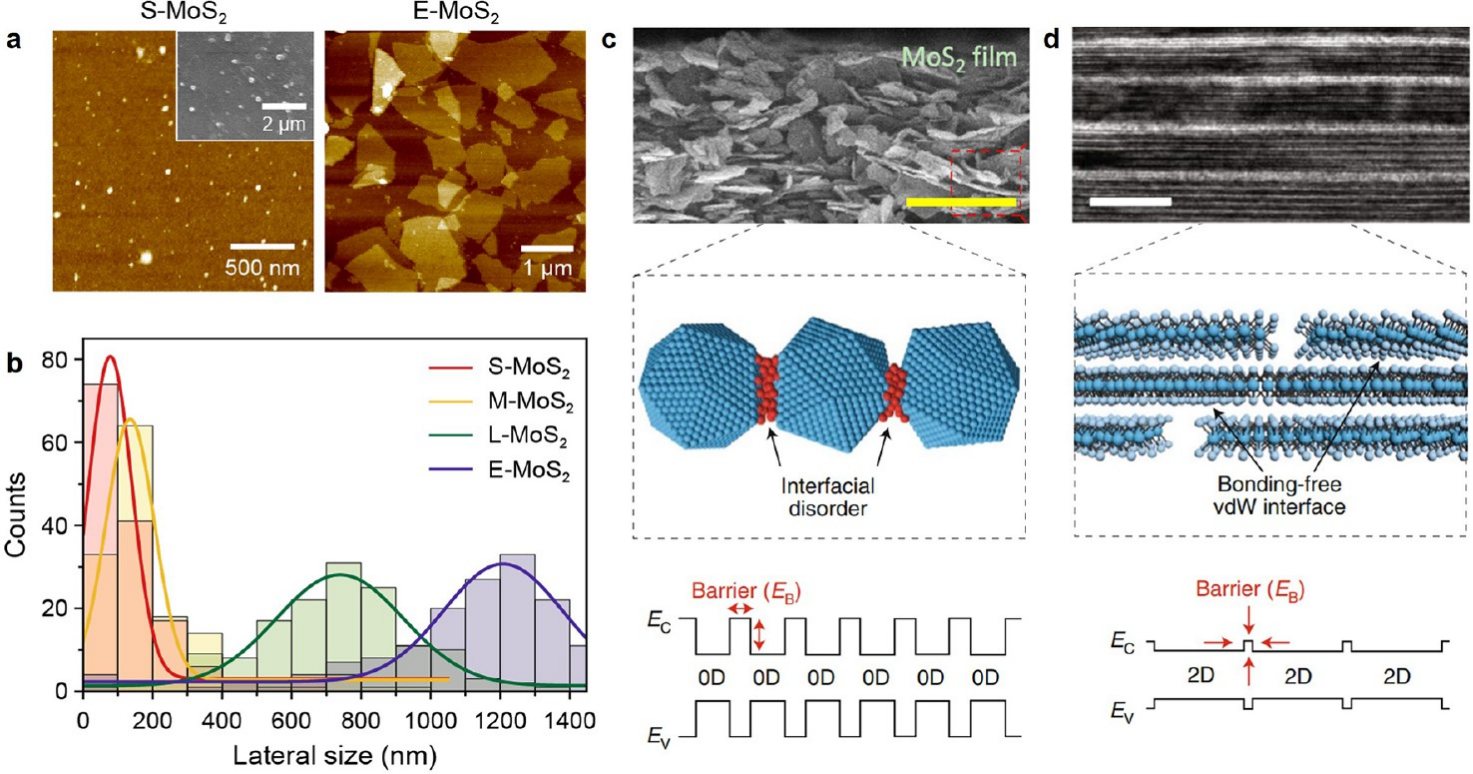
Structural characteristics of exfoliated samples. (a) AFM images of liquid-phase exfoliated (S-MoS_2_) and electrochemically exfoliated (E-MoS_2_) samples. (b) Lateral size distributions of liquid-phase exfoliated (S-, M-, and L-MoS_2_) and E-MoS_2_. Reproduced with permission from ref (48). Copyright 2021 Wiley. (c) Cross-sectional SEM image of assembled liquid-phase exfoliated MoS_2_ film and illustration of interfacial disorder and energy barrier formation. Reproduced from ref (43). Copyright 2021 American Chemical Society. (d) Cross-sectional TEM image of E-MoS_2_ vdW thin film with bonding-free vdW interface-induced minimum contact energy barrier. Reproduced with permission from ref (35). Copyright 2019 Springer.

The resulting structures of the nanosheets produced by direct liquid-phase exfoliation and intercalation-based exfoliation can clearly be distinguished from one another by atomic force microscopic (AFM) analysis of their thickness and lateral size (Figure 2a). Based on their relative lateral size (as controlled by the centrifuge conditions), liquid-phase exfoliated nanosheets are denoted as S- (small), M- (medium), and L-MoS_2_ (large) (Figure 2b).^48^ The larger lateral size is highly desirable for electronic applications, but the liquid-phase exfoliated L-MoS_2_ remains limited due to its broad distribution in thickness. In contrast, molecular intercalation-based electrochemically exfoliated MoS_2_ nanosheets (E-MoS_2_) possess a lateral size (>1 μm) relatively larger than that of liquid-phase exfoliated samples, whereas their thicknesses are atomically thin and highly monodisperse. Larger sizes and narrower thickness distributions become essential when the nanosheets form electronically active thin-film networks. As shown in the cross-sectional microscopic images, point and/or line contacts with interfacial disorder and noticeable energy barriers are dominant between liquid-phase exfoliated samples, while the E-MoS_2_ nanosheets are clearly stacked by forming bonding-free vdW interfaces with a negligible energy barrier (Figures 2c,d).^35,43,49^ Furthermore, the E-MoS_2_-based vdW thin films with broad-area sheet-to-sheet contacts are robust under various mechanical deformations, implying that solution-processed vdW layered materials are strong candidates for flexible and stretchable electronics.^50^

## 3 Electronics Based on Solution-Processed 2D vdW Materials

**Figure 3:**
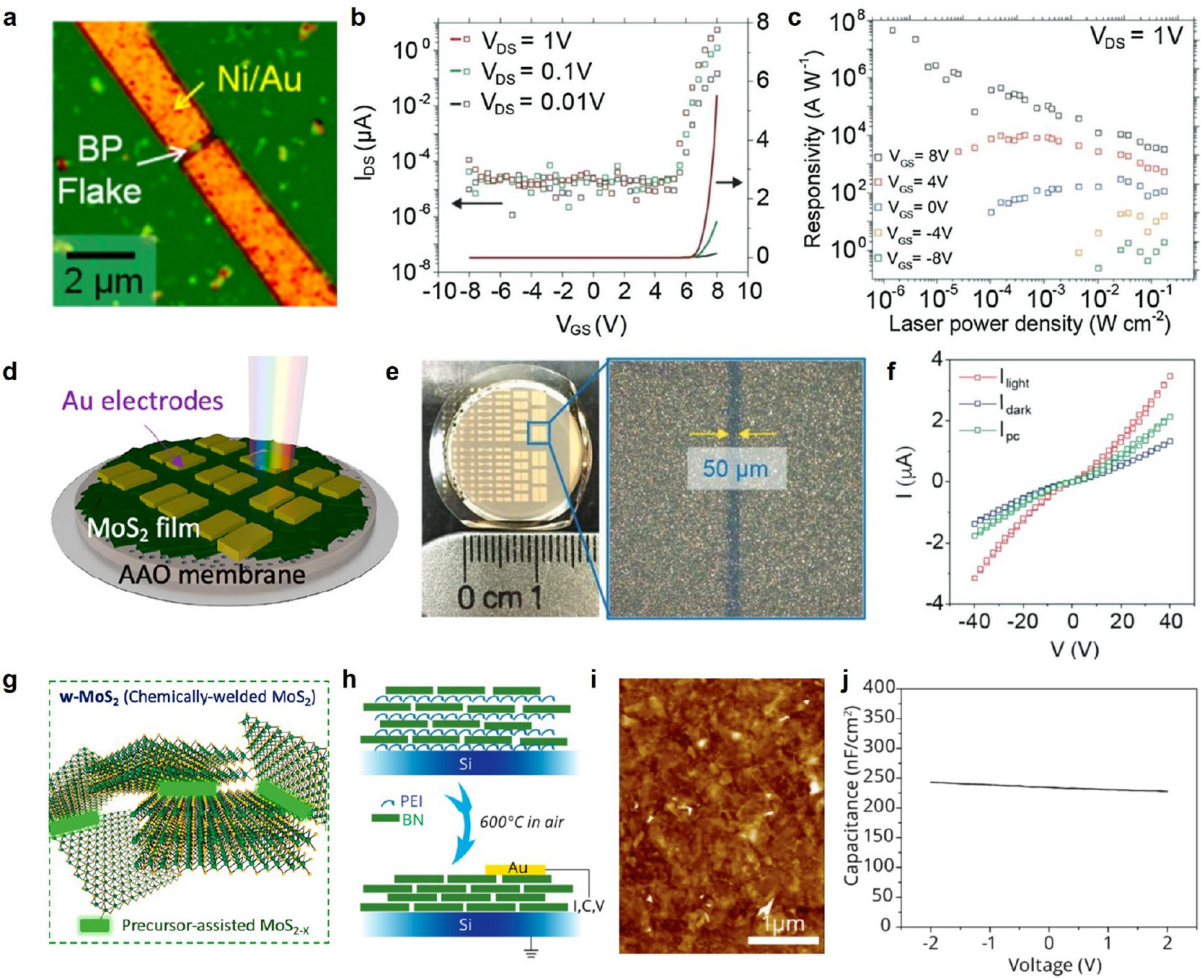
Electronic applications based on liquid-phase exfoliated vdW layered materials. (a) Optical image of a field-effect transistor based on an individual black phosphorus (BP) nanosheet. Reproduced from ref (52). Copyright 2015 American Chemical Society. Gate-dependent (b) electrical and (c) optoelectronic properties of an individual InSe nanosheet. (d) Schematic and (e) photograph of electronically active film prepared by vacuum filtration. (f) Photoresponse of vacuum-filtered InSe film. (g) Conceptual illustration of chemical welding. (h) Layer-by-layer assembled thickness-sorted h-BN for dielectric layer. (i) AFM image of assembled h-BN layers. (j) Capacitance of solution-processed h-BN film. Reproduced from ref (8). Copyright 2015 American Chemical Society. Subpanels (b), (c), (e), and (f) are reproduced with permission from ref (54). Copyright 2018 John Wiley and Sons. Panels (d) and (g) are reproduced from ref (43). Copyright 2021 American Chemical Society.

As discussed above, because the lateral size of nanosheets is directly correlated to the channel length between the source and drain electrodes, there are only limited options for device fabrication based on liquid-phase exfoliated samples with lateral sizes on the order of hundreds of nanometers. For example, the electrical properties of liquid-phase exfoliated individual black phosphorus nanosheets were explored using electron beam lithography (Figure 3a).^51,52^ Similarly, gate-dependent electrical and optoelectronic properties have been reported based on individual InSe nanosheets processed in an additive-free, alcohol–water mixed cosolvent system (Figure 3b,c).^53,54^ In particular, the remarkably high photoresponsivity (∼10^8^ A/W) of liquid-phase exfoliated InSe nanosheets indicates that additive-free liquid-phase exfoliation enables the production of optoelectronically active, high-quality materials on a large scale. Although the quality of various liquid-phase exfoliated individual nanosheets has been verified for use in electronic applications, the demonstration of scalable, gate-tunable high-performance electronics has been hindered by structural limitations including small lateral size and polydisperse thickness. In order to achieve gate-tunable devices, the channel should be electrically conductive but thin enough to be fully depleted via gate-dependent electrostatic doping over the entire channel. As shown in Figure 2c, the structural polydispersity-induced randomly stacked structures are unable to sufficiently satisfy both the specified thin-film thickness and electrical contacts in the in-plane direction. Consequently, the demonstration of scalable electronics based on liquid-phase exfoliated 2D nanosheets has been limited mainly to two-terminal conductor-type devices.^53−55^ For example, a controlled amount of 2D material dispersion can be collected on a porous membrane such as anodic alumina (AAO) to form an electrically active percolated network structure, followed by electrode array deposition (Figure 3d,e). As shown in Figure 3f, this array can be utilized as a photoconductor by collecting the photocurrent (*I*_pc_ = *I*_light_ – *I*_dark_) under light illumination. To minimize the contact resistance of films originating from inefficient sheet-to-sheet contact, a chemical welding approach has been developed to convert a liquid-phase precursor, ammonium tetrathiomolybdate, to MoS_2_ at the interfaces under heat treatment.^43^ This approach significantly improves electrical conduction through the MoS_2_ film, while providing additional functionality associated with thermally controllable sulfur vacancies; however, the overall film thickness still needs to be optimized to achieve gate-tunable devices (Figure 3g). A liquid-phase exfoliated insulator, h-BN, has also been utilized as a dielectric layer in graphene-based field-effect transistors (GFETs)—following precise layer sorting via isopycnic density gradient ultracentrifugation. A well-packed dielectric thin film was prepared from layer-by-layer assembly of alternately stacked polyethylenimine (PEI) (a charged polymer) and thickness-sorted h-BN nanosheets (Figure 3h). Layer-by-layer assembly has been intensively utilized to form a percolating network based on solution-processed nanomaterials. In principle, this method is a self-limiting nanoscale additive manufacturing technique to assemble into thickness-controlled thin films by alternating exposure of electrostatically attractive two or more species.^56^ Following an additional annealing process, the resulting h-BN thin-film network exhibited low leakage currents and high capacitances, further enabling hysteresis-free, high-mobility GFETs (Figure 3i,j).^8,11^

**Figure 4:**
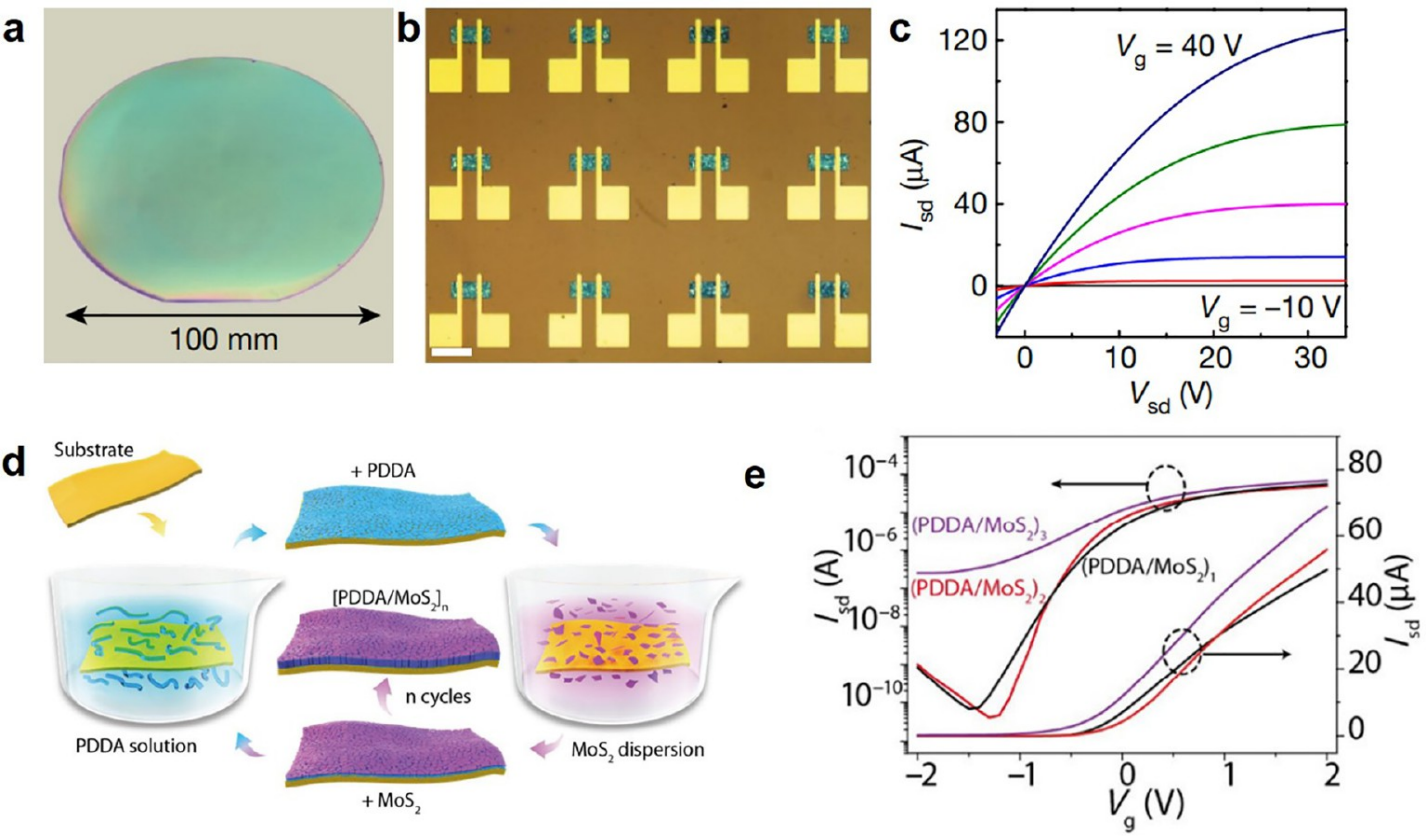
Gate-tunable electronic properties on electrochemically exfoliated vdW layered materials. (a) Wafer-scale deposition of electrochemically exfoliated MoS_2_ nanosheets. (b) Field-effect transistor array on MoS_2_ vdW thin film. (c) Gate-dependent output characteristics. Reproduced with permission from ref (36). Copyright 2018 Springer. (d) Schematic of layer-by-layer assembly of MoS_2_ for vdW thin-film formation. (e) Gate-dependent transfer characteristics. Reproduced with permission from ref (56). Copyright 2021 Springer.

In contrast to the limited electronic applications of liquid-phase exfoliated 2D nanosheets, the molecular intercalation-based electrochemical exfoliation approach enables the feasibility of solution-based approaches for wafer-scale electronics.^36^ Most importantly, atomically thin, highly monodisperse in thickness, and relatively large (∼1 μm) nanosheet structures enable the formation of compact thin films simply by spin coating, as shown in Figure 4a. These nanosheet building blocks form thin-film networks with thicknesses on the order of nanometers and vdW interfaces over a 100 mm diameter SiO_2_/Si wafer—a potential precursor for high-performance thin-film electronics on a rigid substrate (Figure 4b).^36^ The output characteristic demonstrates that the resulting MoS_2_ thin film can be electrostatically modulated through the application of a gate voltage (Figure 4c). The favorable structure of the individual nanosheet further enables the use of conventional layer-by-layer assembly, as presented in Figure 3h, to obtain scalable thin films by alternative exposure to exfoliated MoS_2_ nanosheets and cationic poly(diallyl dimethylammonium chloride, PDDA) for electrostatic interactions (Figure 4d).^56^ To optimize the performance of the electronic device, the semiconducting channel thickness can be controlled through repeated coating iterations. As a result, both spin-coated and layer-by-layer assembled MoS_2_ thin films functioned as high-performance thin-film transistors with field-effect mobilities of ∼10 cm^2^/(V s) at room temperature and a current on/off ratio of 10^6^ (Figure 4e), which is comparable to the performance of individual MoS_2_ nanosheets.^36,56^

**Figure 5:**
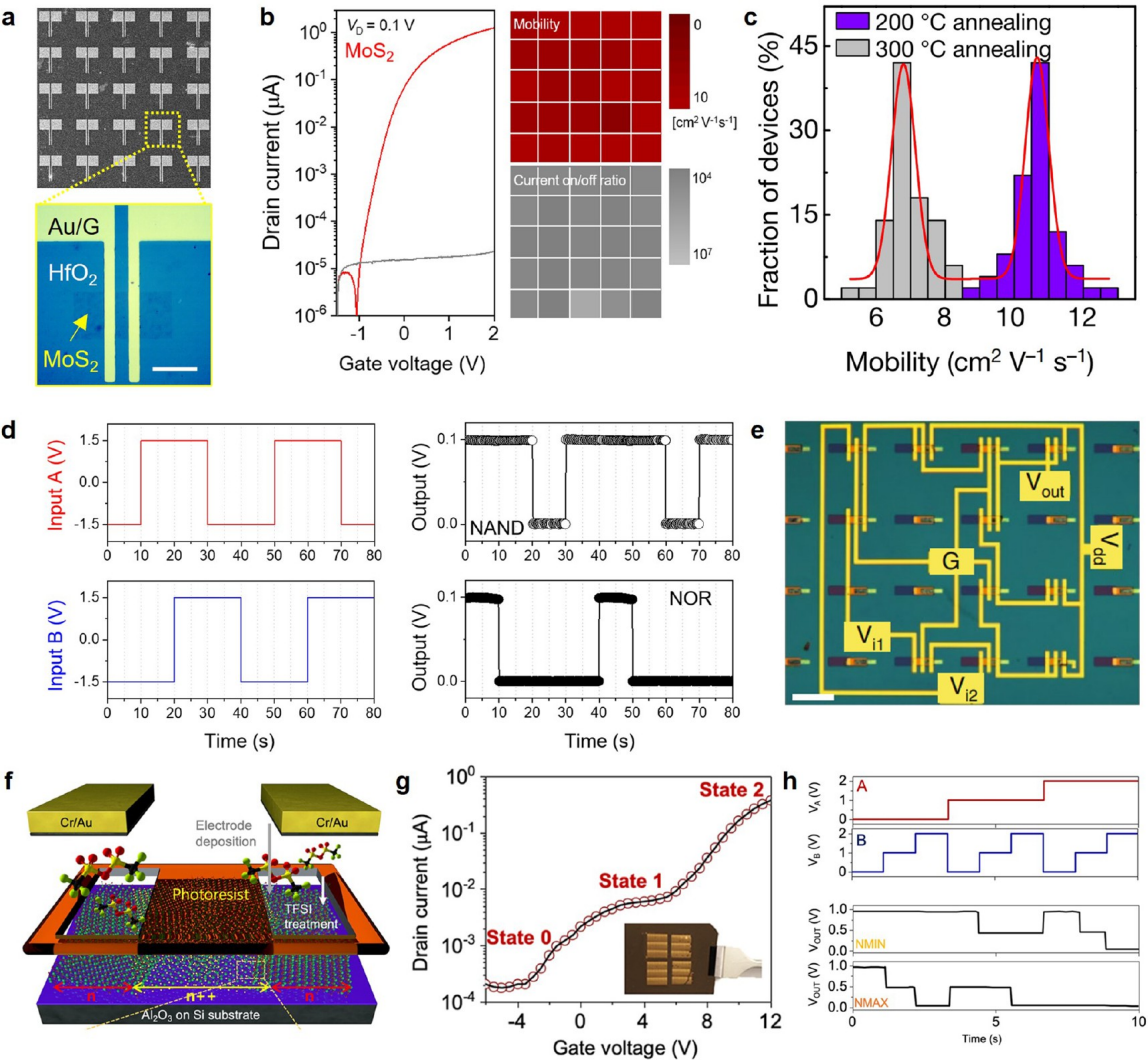
Spatial uniformity of vdW thin films and logic-gate applications. (a) Field-effect transistor array on electrochemically exfoliated MoS_2_ vdW thin films and (b) corresponding electronic characteristics. Reproduced with permission from ref (57). Copyright 2022 John Wiley and Sons. (c) Thermal annealing-dependent field-effect mobility distributions. Reproduced with permission from ref (36). Copyright 2018 Springer. Implemented logic gates including (d) NAND and NOR and (e) XOR. (f) Structure of ternary device based on area-selective chemical doping. (g) Representative ternary transfer characteristic and (h) NMIN and NMAX logic gates. Reproduced from ref (59). Copyright 2022 American Chemical Society.

In addition, vdW thin films based on electrochemically exfoliated nanosheets exhibit remarkably high spatial uniformity, resulting in a high device production yield (>95%)^36^ enabling the synthesis of higher complexity devices, including logic gates. A scanning electron microscopic image of a transistor array fabricated on a MoS_2_ vdW thin film is shown in Figure 5a.^57^ A representative transfer characteristic is shown with spatial mapping of highly uniform field-effect mobility (red) and current on/off ratio (gray) (Figure 5b). The field-effect mobility of each device can be further optimized by manipulating the annealing temperature; the narrow distribution of the resulting histogram indicates that the vdW thin film is electronically active with high spatial uniformity (Figure 5c), which enables the demonstrations of logic gates such as NAND, NOR, and XOR, as shown in Figure 5d,e.^36,57^ Furthermore, multivalued logic gates have been implemented with MoS_2_ vdW thin films by area-selective chemical doping (Figure 5f). A representative transfer curve of the ternary transistor is shown in Figure 5g, where an intermediate state (state 1) placed between the off (state 0) and on (state 2) states is induced by the energy-band misalignment of the doped/undoped MoS_2_ channel regions. Ternary devices have also been implemented to demonstrate multivalued logic gates, such as NMIN and NMAX (Figure 5h) at the wafer scale.^58,59^

**Figure 6:**
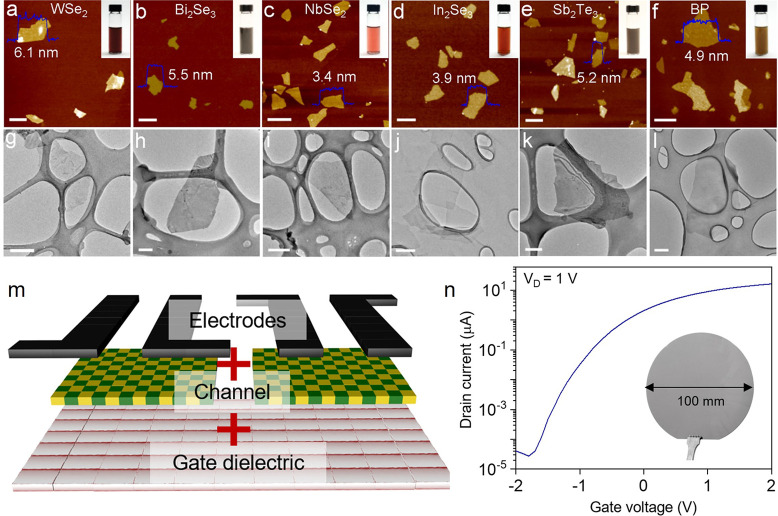
Universal approach to electrochemical exfoliation for potential vdW heterostructures. (a–l) AFM and TEM images of vdW layered materials synthesized via electrochemical exfoliation including WSe_2_, Bi_2_Se_3_, NbSe_2_, In_2_Se_3_, Sb_2_Te_3_, and BP. Reproduced with permission from ref (36). Copyright 2018 Springer. (m) Schematic of all vdW layered materials-based heterostructure and (n) wafer-scale implementation of all 2D-based field-effect transistors. Reproduced with permission from ref (57). Copyright 2022 John Wiley and Sons.

The successful development of various electronic devices based on solution-processed MoS_2_ vdW thin films as semiconducting channels has motivated the synthesis of other structurally analogous materials with various electrical properties. For example, the approaches described in previous sections are also applicable to alternative layered bulk crystals such as WSe_2_, Bi_2_Se_3_, NbSe_2_, In_2_Se_3_, Sb_2_Te_3_, and black phosphorus (BP).^36^ The representative structures of each sample are illustrated in Figure 6a–f (AFM images) and Figure 6g–l (transmission electron microscopy (TEM) images). These atomically thin 2D materials possess a reasonably large lateral size and can therefore be implemented in electronic devices such as electrodes, semiconducting channels, and dielectric layers, which is consistent with their electrical properties. Furthermore, two or more materials can be assembled into a single electronic device, as illustrated in Figure 6m. For example, wafer-scale vdW heterostructures were synthesized from laterally and vertically stacked metallic graphene, semiconducting MoS_2_, and insulating HfO_2_ vdW thin films as electrodes, channels, and gate dielectrics, respectively (Figure 6n).^57^

## 4 Assembly of Solution-Processed vdW Layered Materials

To fully exploit solution-processed vdW layered materials for scalable applications, many deposition or wet-transfer methods have been introduced to form electronically active vdW thin-film networks based on stable dispersion. The above-mentioned deposition methods (i.e., vacuum filtration, spin coating, and layer-by-layer assembly) and other commonly used thin-film deposition techniques (e.g., spray coating, bar coating, and Langmuir–Blodgett) can successfully generate vdW thin films for electronic device fabrication. In addition to the direct deposition, wet-transfer methods have been utilized for many purposes including changing substrates,^60^ transmission electron microscopic analysis,^61^ and multilayered heterostructure formation.^62^ For the transfer, in general, the poly(methyl methacrylate) (PMMA) layer is coated on any 2D samples exfoliated/grown on an arbitrary substrate; the substrate is removed by etchant exposure; the PMMA/2D layer is carefully transferred onto a target substrate, and PMMA is removed. This wet-transfer method is applicable not only for vdW material dispersions but also micromechanically exfoliated or chemically grown samples.

**Figure 7:**
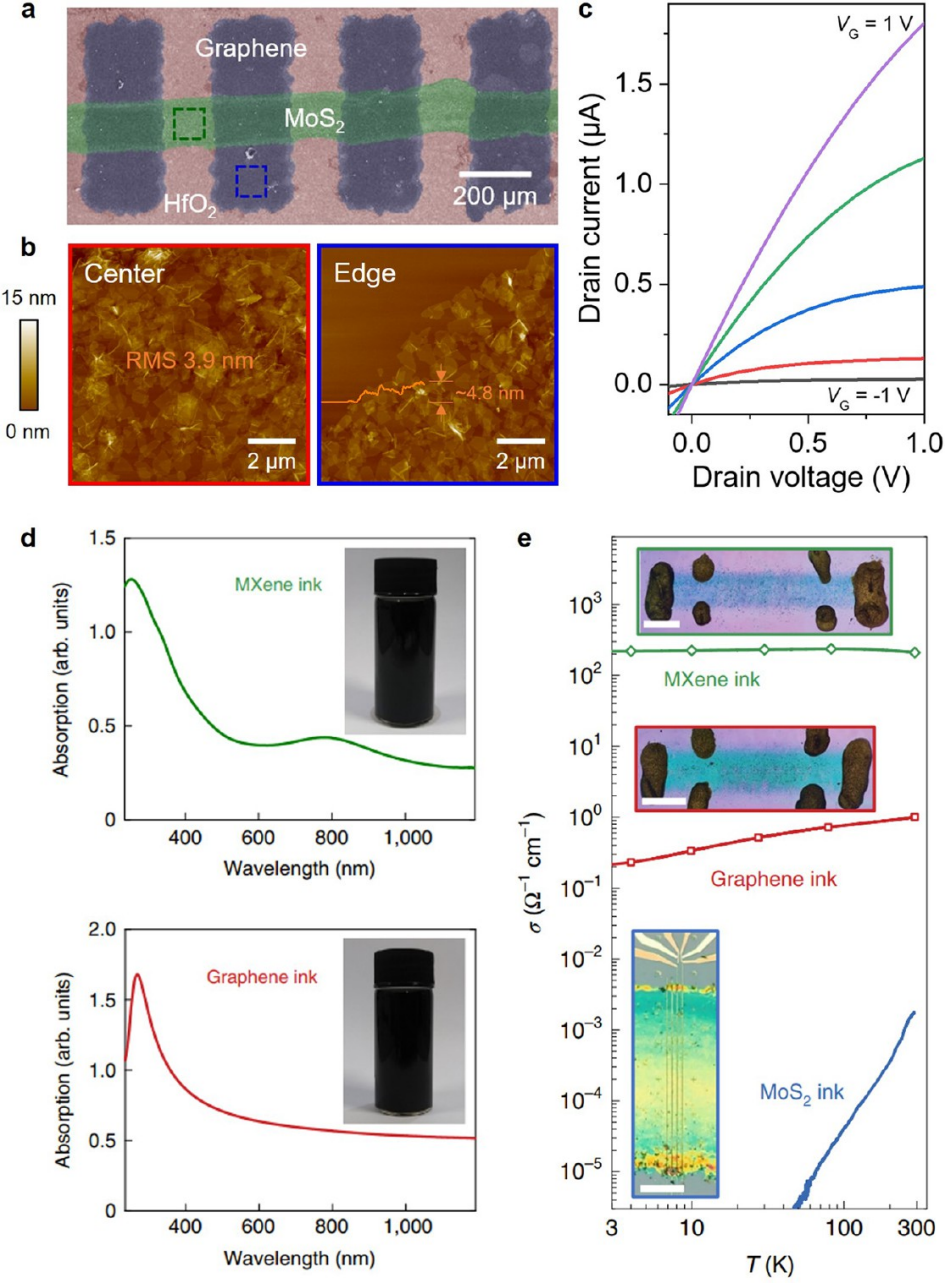
All inkjet-printed thin-film electronics based on vdW layered materials. (a) False-color optical image of all vdW layered materials-based field-effect transistor. (b) AFM images of center and edge of the printed pattern. (c) Gate-dependent output characteristic of printed transistor. Reproduced with permission from ref (57). Copyright 2022 John Wiley and Sons. (d) Absorbance spectra of MXene and graphene inks for inkjet-printed electrodes. (e) Temperature-dependent electrical properties. Reproduced with permission from ref (10). Copyright 2021 Springer.

Printing technology has also been extensively developed following successful 2D ink formulations.^10,34,57,63,64^ Printing-based assembly is highly cost-effective due to its inherent waste minimization; desired structures can be achieved in the mask- and vacuum-free process. Among the various printing approaches, inkjet printing is the most accessible for the development of electronic device prototypes from 2D material inks. To successfully implement printed electronic devices, 2D material inks should satisfy several criteria. First, 2D materials should be adequately stabilized with respect to aggregation (for a reasonable period of time) to support inkjet printing with high device-to-device reliability. In addition to ink stability, the rheological properties of the inks (including viscosity, surface tension, and density, where the overall relationship is defined as the inverse Ansorge number) also factor in considerably to stable ink jetting. Finally, the structural properties of 2D nanosheets, such as thickness, lateral size, and the concentration of the ink, should be optimized to avoid clogging of the nozzle during printing. The development and optimization of 2D material inks have enabled the prototyping of several printed electronic devices, such as field-effect transistors, photodetectors, memory devices, and logic gates.^10,34,57,63,64^ For example, as shown in Figure 7a, various 2D materials with different electronic types have been sequentially printed for integration into a single functional device, where metallic graphene, semiconducting MoS_2_, and insulating HfO_2_ are used as electrodes, semiconductor channels, and dielectric layers, respectively. The AFM images exhibits the stacking morphology of printed 2D nanosheets at the center and edge of the pattern (Figure 7b).^57^ The resulting all inkjet-printed field-effect transistor device exhibits gate-tunable current–voltage modulations, which indicate that the inkjet-printed MoS_2_ channel is sufficiently thin, while remaining electrically conductive to enable gate modulation (Figure 7c). To further elucidate the charge transport mechanisms of inkjet-printed 2D nanosheets, the electrical conductivity of the printed 2D thin-film networks was explored at various temperatures, levels of gate modulation, and magnetic field. In addition to semimetallic graphene, titanium carbide (Ti_3_C_2_) MXene can also be exploited as a stable ink for use in conductive thin-film networks as electrodes.^10^ The optical absorbance spectra of the as-synthesized MXene and graphene inks are shown in Figure 7d, which enables the straightforward evaluation of concentration and ink dispersion stability. Following inkjet printing of the prepared stable inks, the temperature dependence of electrical conductivity has also been explored with respect to inkjet-printed MXene, graphene, and MoS_2_ thin-film networks. Thorough investigation of the temperature-, gate-modulation-, and magnetic-field-dependent electrical properties revealed that the charge transport mechanism of each material is dominated by intrinsic intra-nanosheet or inter-nanosheet processes. For example, the charge transport of a printed graphene thin film is governed by nanosheet-to-nanosheet contacts, resulting in variable-range hopping (VRH) mechanisms, while printed MXene and MoS_2_ thin films show a temperature-dependent transition of VRH to the nearest-neighbor hopping mechanism because of the dominant intrananosheet charge transport (Figure 7e).^10^

## 5 Scalable Demonstrations of Flexible Electronics

**Figure 8:**
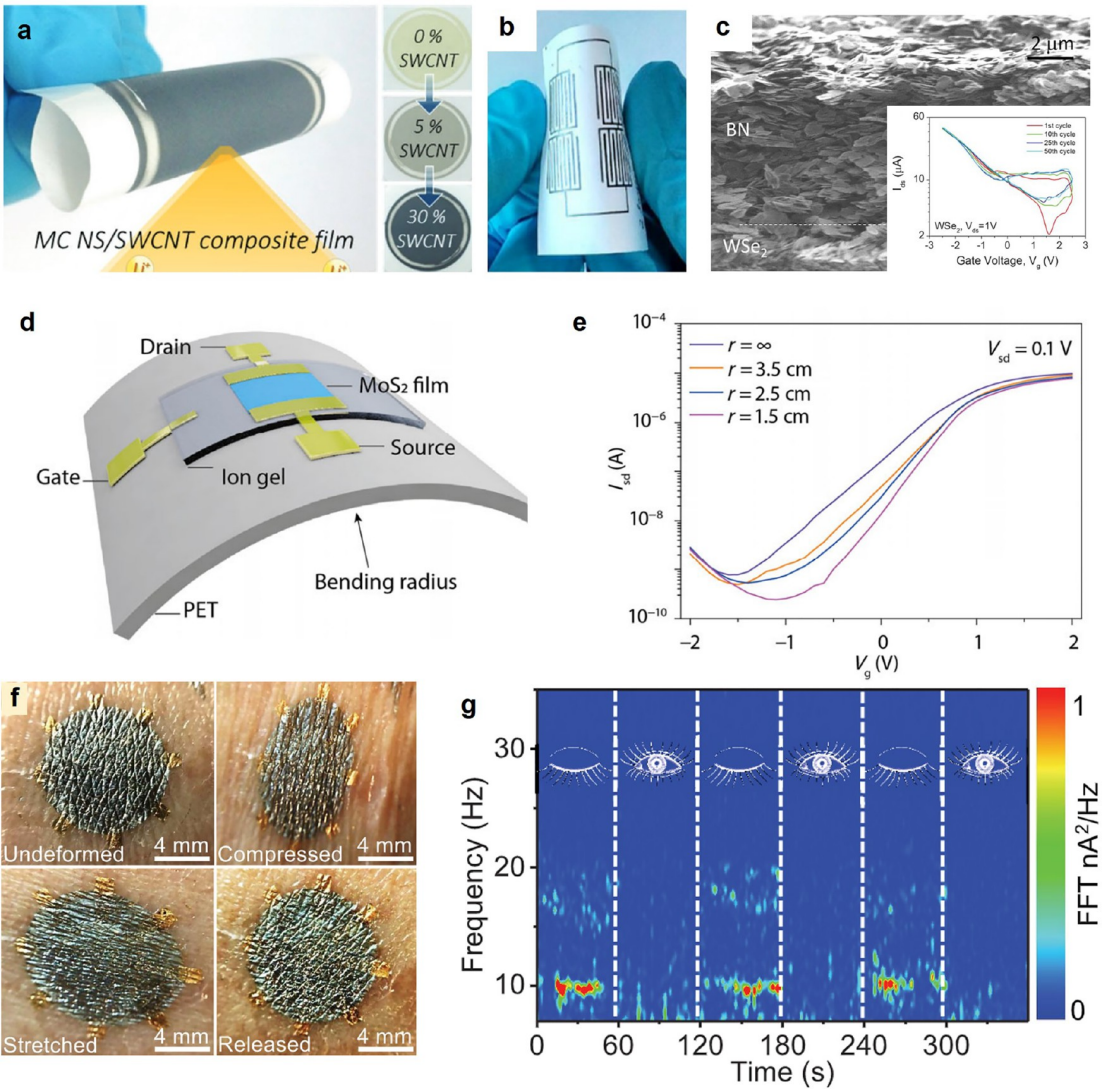
Mechanically deformable electronics based on solution-processed vdW thin films. (a) Foldable lithium-ion battery anode applications based on vdW nanosheet and CNT composite films. Reproduced with permission from ref (65). Copyright 2017 John Wiley and Sons. (b) Printed MXene electrodes for flexible microsupercapacitors. Reproduced with permission under a Creative Commons CC BY license from ref (66) Copyright 2019 The Authors. (c) All inkjet printed gate-tunable vdW transistors. Reproduced with permission from ref (63). Copyright 2017 AAAS. (d) Schematic and (e) transfer characteristics of vdW thin-film transistors on a flexible substrate with controlled bending radius. Reproduced with permission from ref (56). Copyright 2021 Springer. (f) Photographs of vdW thin film on human skin under various mechanical deformations. (g) Time-dependent signal recorded during cyclic eye closing and opening. Reproduced with permission from ref (50). Copyright 2022 AAAS.

Finally, solution-processed 2D nanosheet-based continuous films were utilized for the scalable implementation of flexible electronic applications. 2D nanosheet thin films prepared by direct liquid-phase exfoliation have mainly been applied as metallic elements in electronic applications such as electrodes. To maintain their mechanical stability while the flexible devices are in operation, a range of heterogeneous composites have also been explored; for example, liquid-phase exfoliated 2D nanosheets mixed in a 1D carbon nanotube percolated network have been utilized as a flexible electrode for potential wearable energy storage applications (Figure 8a).^65^ In addition, metallic MXene inks can be printed as interdigitated electrodes on flexible substrates, such as porous paper and aluminum foil, for use in micro-supercapacitors (Figure 8b).^66^ Beyond the use of liquid-phase exfoliated 2D nanosheets as metallic elements, 2D materials with various electronic properties have been inkjet-printed onto plastic to demonstrate a flexible thin-film transistor array, where graphene, WSe_2_, and h-BN nanosheets are sequentially printed as the electrodes, semiconducting channel, and dielectric layer, respectively (Figure 8c). Although this transistor array offers gate tunability through the use of an ionic liquid to facilitate electrolytic gate modulation, the device performance is still limited as current on/off ratios and field-effect mobility remain at ∼25 and ∼0.1 cm^2^/(V s) (inset of Figure 8c), respectively, resulting primarily from the randomly disordered nanosheet-to-nanosheet interfaces of liquid-phase exfoliated samples.^63^ However, the device performance of thin-film transistors has been significantly improved even under bending conditions on a flexible substrate through the development of electrochemically exfoliated 2D nanosheets (Figure 8d,e).^56^ This can be attributed primarily to the high structural uniformity of the electrochemically exfoliated 2D individual nanosheets and the thin-film networks formed by vdW contacts. The effectively formed plane-to-plane contacts in thin-film networks allow mechanical stability under local tension or compression without losing conductive pathways.^50^ This remarkable mechanical stability enables the extension of 2D vdW thin-film-based electronics into bioelectronic applications through integration with soft objects such as human skin and leaves. As shown in Figure 8f, the vdW thin film exhibits conformal contact with the skin while it is repeatedly squeezed and stretched. As a result, the electrical signal of the thin film is stable under mechanical deformations, which further enables the implementation of thin-film devices for monitoring transient potentials such as cyclic eye closing and opening (Figure 8g).^50^

## 6 Conclusion and Outlook

Having achieved significant breakthroughs in the development of electrochemically driven molecular intercalation-based exfoliation of layered crystals, the solution-based production of 2D vdW materials can take us step closer to mass production for practical applications. Although many promising prototype studies have been demonstrated in the domain of electronics based on these samples, several challenges remain to fully exploit their potential. First, layered crystals are not always suitable for application in electrochemical exfoliation. To initiate the electrochemical reaction, bulk samples (either natural crystals or those grown by chemical vapor transport) should be electrically conductive. Therefore, several conductive materials have been successfully exfoliated, including metallic graphene and semiconducting MoS_2_, InSe, and BP, while insulating materials have not been directly applicable. As alternatives, layer separation of liquid-phase exfoliated hexagonal boron nitride and electrochemical exfoliation of semiconducting HfS_2_ nanosheets followed by thermal oxidation have been introduced to produce insulating nanosheets.^8,57^ Although these approaches enable the preparation of ultrathin dielectric layers for electronic applications, additional processes such as isopycnic density gradient ultracentrifugation or high-temperature thermal annealing are also required. Second, processing-induced defect generation can also be a challenging issue in maintaining the intrinsic electronic properties. For example, the formation of chalcogen (S or Se) vacancies in conjunction with the exfoliation of transition metal dichalcogenides results in heavily n-doped electronic behavior, which requires post-treatment to reduce the chalcogen vacancy concentration. In this regard, a nonoxidizing organic superacid (e.g., bis(trifluoromethane) sulfonimide) treatment has been introduced to minimize sulfur vacancies on MoS_2_ nanosheets, resulting in significantly reduced off-current as well as highly improved photoluminescence intensity.^36,57,59,67^ However, such chemical treatment approaches for other chalcogen vacancies are still missing. Chemical oxidation is another potential issue to address to extend the processing of ambient reactive materials (e.g., BP and InSe).^51,52,54^ To minimize this issue, the exfoliation process should be carefully performed under controlled conditions in anhydrous solvents in an O_2_ and/or water-free environment. Third, both the synthesis of high-quality 2D material dispersions and electronic device fabrication also require the full exploitation of solution-based processing. The previously reported MoS_2_-based state-of-the-art thin-film transistors showed an average field-effect mobility of 10 cm^2^/(V s), which lags far behind the promising device performance of other future electronics. For device fabrication, the properties of 2D ink formulations are enable the use of printing-based, lithography-free techniques. However, current printing technologies should be further improved to achieve minimum patternable resolution and spatial accuracy to precisely form heterogeneous 2D materials into the desired device structures. Consequently, the development of the electrochemical exfoliation of 2D materials has introduced a new frontier into solution-based processing for scalable electronic applications; however, there remain significant challenges and opportunities which must be navigated in realizing their potential in next-generation electronics.

## References

[ref1] Novoselov K. S.; Jiang D.; Schedin F.; Booth T. J.; Khotkevich V. V.; Morozov S. V.; Geim A. K. Two-dimensional atomic crystals. Proc. Natl. Acad. Sci. U.S.A. 2005, 102, 10451–10453. 10.1073/pnas.0502848102. 16027370 PMC1180777

[ref2] Geim A. K.; Novoselov K. S. The rise of graphene. Nat. Mater. 2007, 6, 183–191. 10.1038/nmat1849. 17330084

[ref3] Dean C. R.; Young A. F.; Meric I.; Lee C.; Wang L.; Sorgenfrei S.; Watanabe K.; Taniguchi T.; Kim P.; Shepard K. L.; Hone J. Boron nitride substrates for high-quality graphene electronics. Nat. Nanotechnol. 2010, 5, 722–726. 10.1038/nnano.2010.172. 20729834

[ref4] Jariwala D.; Sangwan V. K.; Lauhon L. J.; Marks T. J.; Hersam M. C. Emerging Device Applications for Semiconducting Two-Dimensional Transition Metal Dichalcogenides. ACS Nano 2014, 8, 1102–1120. 10.1021/nn500064s. 24476095

[ref5] Wang Q. H.; Kalantar-Zadeh K.; Kis A.; Coleman J. N.; Strano M. S. Electronics and optoelectronics of two-dimensional transition metal dichalcogenides. Nat. Nanotechnol. 2012, 7, 699–712. 10.1038/nnano.2012.193. 23132225

[ref6] Li L. K.; Yu Y. J.; Ye G. J.; Ge Q. Q.; Ou X. D.; Wu H.; Feng D. L.; Chen X. H.; Zhang Y. B. Black phosphorus field-effect transistors. Nat. Nanotechnol. 2014, 9, 372–377. 10.1038/nnano.2014.35. 24584274

[ref7] Ryder C. R.; Wood J. D.; Wells S. A.; Hersam M. C. Chemically Tailoring Semiconducting Two-Dimensional Transition Metal Dichalcogenides and Black Phosphorus. ACS Nano 2016, 10, 3900–3917. 10.1021/acsnano.6b01091. 27018800

[ref8] Zhu J.; Kang J.; Kang J. M.; Jariwala D.; Wood J. D.; Seo J. W. T.; Chen K. S.; Marks T. J.; Hersam M. C. Solution-Processed Dielectrics Based on Thickness-Sorted Two-Dimensional Hexagonal Boron Nitride Nanosheets. Nano Lett. 2015, 15, 7029–7036. 10.1021/acs.nanolett.5b03075. 26348822

[ref9] Bandurin D. A.; Tyurnina A. V.; Yu G. L.; Mishchenko A.; Zolyomi V.; Morozov S. V.; Kumar R. K.; Gorbachev R. V.; Kudrynskyi Z. R.; Pezzini S.; Kovalyuk Z. D.; Zeitler U.; Novoselov K. S.; Patane A.; Eaves L.; Grigorieva I. V.; Fal’ko V. I.; Geim A. K.; Cao Y. High electron mobility, quantum Hall effect and anomalous optical response in atomically thin InSe. Nat. Nanotechnol. 2017, 12, 223 10.1038/nnano.2016.242. 27870843

[ref10] Piatti E.; Arbab A.; Galanti F.; Carey T.; Anzi L.; Spurling D.; Roy A.; Zhussupbekova A.; Patel K. A.; Kim J. M.; Daghero D.; Sordan R.; Nicolosi V.; Gonnelli R. S.; Torrisi F. Charge transport mechanisms in inkjet-printed thin-film transistors based on two-dimensional materials. Nat. Electron. 2021, 4, 893–905. 10.1038/s41928-021-00684-9.

[ref11] Zhu J.; Liu X. L.; Geier M. L.; McMorrow J. J.; Jariwala D.; Beck M. E.; Huang W.; Marks T. J.; Hersam M. C. Layer-by-Layer Assembled 2D Montmorillonite Dielectrics for Solution-Processed Electronics. Adv. Mater. 2016, 28, 63–68. 10.1002/adma.201504501. 26514248

[ref12] Gogotsi Y.; Anasori B. The Rise of MXenes. ACS Nano 2019, 13 (8), 8491–8494. 10.1021/acsnano.9b06394. 31454866

[ref13] Kim H.; Alshareef H. N. MXetronics: MXene-Enabled Electronic and Photonic Devices. Acs Mater. Lett. 2020, 2, 55–70. 10.1021/acsmaterialslett.9b00419.

[ref14] Kim S.; Jo S. B.; Kim J.; Rhee D.; Choi Y. Y.; Kim D. H.; Kang J.; Cho J. H. Gate-Deterministic Remote Doping Enables Highly Retentive Graphene-MXene Hybrid Memory Devices on Plastic. Adv. Funct. Mater. 2022, 2111956 10.1002/adfm.202111956.

[ref15] Fiori G.; Bonaccorso F.; Iannaccone G.; Palacios T.; Neumaier D.; Seabaugh A.; Banerjee S. K.; Colombo L. Electronics based on two-dimensional materials. Nat. Nanotechnol. 2014, 9, 768–779. 10.1038/nnano.2014.207. 25286272

[ref16] Wang X. R.; Dai H. J. Etching and narrowing of graphene from the edges. Nat. Chem. 2010, 2, 661–665. 10.1038/nchem.719. 20651729

[ref17] Cassabois G.; Valvin P.; Gil B. Hexagonal boron nitride is an indirect bandgap semiconductor. Nat. Photonics 2016, 10 (4), 262 10.1038/nphoton.2015.277.

[ref18] Lee C. H.; Lee G. H.; van der Zande A. M.; Chen W. C.; Li Y. L.; Han M. Y.; Cui X.; Arefe G.; Nuckolls C.; Heinz T. F.; Guo J.; Hone J.; Kim P. Atomically thin p–n junctions with van der Waals heterointerfaces. Nat. Nanotechnol. 2014, 9, 676–681. 10.1038/nnano.2014.150. 25108809

[ref19] Geim A. K.; Grigorieva I. V. Van der Waals heterostructures. Nature 2013, 499, 419–425. 10.1038/nature12385. 23887427

[ref20] Jariwala D.; Marks T. J.; Hersam M. C. Mixed-dimensional van der Waals heterostructures. Nat. Mater. 2017, 16, 170–181. 10.1038/nmat4703. 27479211

[ref21] Liu Y.; Weiss N. O.; Duan X. D.; Cheng H. C.; Huang Y.; Duan X. F. Van der Waals heterostructures and devices. Nat. Rev. Mater. 2016, 1, 16042 10.1038/natrevmats.2016.42.

[ref22] Novoselov K. S.; Mishchenko A.; Carvalho A.; Castro Neto A. H. 2D materials and van der Waals heterostructures. Science 2016, 353, 461 10.1126/science.aac9439. 27471306

[ref23] Liang S. J.; Cheng B.; Cui X. Y.; Miao F. Van der Waals Heterostructures for High-Performance Device Applications: Challenges and Opportunities. Adv. Mater. 2019, 32, 1903800 10.1002/adma.201903800. 31608514

[ref24] Britnell L.; Gorbachev R. V.; Jalil R.; Belle B. D.; Schedin F.; Mishchenko A.; Georgiou T.; Katsnelson M. I.; Eaves L.; Morozov S. V.; Peres N. M. R.; Leist J.; Geim A. K.; Novoselov K. S.; Ponomarenko L. A. Field-Effect Tunneling Transistor Based on Vertical Graphene Heterostructures. Science 2012, 335, 947–950. 10.1126/science.1218461. 22300848

[ref25] Britnell L.; Gorbachev R. V.; Geim A. K.; Ponomarenko L. A.; Mishchenko A.; Greenaway M. T.; Fromhold T. M.; Novoselov K. S.; Eaves L. Resonant tunnelling and negative differential conductance in graphene transistors. Nat. Commun. 2013, 4, 1794 10.1038/ncomms2817. 23653206 PMC3644101

[ref26] Mishchenko A.; Tu J. S.; Cao Y.; Gorbachev R. V.; Wallbank J. R.; Greenaway M. T.; Morozov V. E.; Morozov S. V.; Zhu M. J.; Wong S. L.; Withers F.; Woods C. R.; Kim Y. J.; Watanabe K.; Taniguchi T.; Vdovin E. E.; Makarovsky O.; Fromhold T. M.; Fal’ko V. I.; Geim A. K.; Eaves L.; Novoselov K. S. Twist-controlled resonant tunnelling in graphene/boron nitride/graphene heterostructures. Nat. Nanotechnol. 2014, 9, 808–813. 10.1038/nnano.2014.187. 25194946

[ref27] Yu W. J.; Li Z.; Zhou H. L.; Chen Y.; Wang Y.; Huang Y.; Duan X. F. Vertically stacked multi-heterostructures of layered materials for logic transistors and complementary inverters. Nat. Mater. 2013, 12, 246–252. 10.1038/nmat3518. 23241535 PMC4249642

[ref28] Backes C.; Higgins T. M.; Kelly A.; Boland C.; Harvey A.; Hanlon D.; Coleman J. N. Guidelines for Exfoliation, Characterization and Processing of Layered Materials Produced by Liquid Exfoliation. Chem. Mater. 2017, 29, 243–255. 10.1021/acs.chemmater.6b03335.

[ref29] Coleman J. N.; Lotya M.; O’Neill A.; Bergin S. D.; King P. J.; Khan U.; Young K.; Gaucher A.; De S.; Smith R. J.; Shvets I. V.; Arora S. K.; Stanton G.; Kim H. Y.; Lee K.; Kim G. T.; Duesberg G. S.; Hallam T.; Boland J. J.; Wang J. J.; Donegan J. F.; Grunlan J. C.; Moriarty G.; Shmeliov A.; Nicholls R. J.; Perkins J. M.; Grieveson E. M.; Theuwissen K.; McComb D. W.; Nellist P. D.; Nicolosi V. Two-Dimensional Nanosheets Produced by Liquid Exfoliation of Layered Materials. Science 2011, 331, 568–571. 10.1126/science.1194975. 21292974

[ref30] Nicolosi V.; Chhowalla M.; Kanatzidis M. G.; Strano M. S.; Coleman J. N. Liquid Exfoliation of Layered Materials. Science 2013, 340, 1420 10.1126/science.1226419.

[ref31] Paton K. R.; Varrla E.; Backes C.; Smith R. J.; Khan U.; O’Neill A.; Boland C.; Lotya M.; Istrate O. M.; King P.; Higgins T.; Barwich S.; May P.; Puczkarski P.; Ahmed I.; Moebius M.; Pettersson H.; Long E.; Coelho J.; O’Brien S. E.; McGuire E. K.; Sanchez B. M.; Duesberg G. S.; McEvoy N.; Pennycook T. J.; Downing C.; Crossley A.; Nicolosi V.; Coleman J. N. Scalable production of large quantities of defect-free few-layer graphene by shear exfoliation in liquids. Nat. Mater. 2014, 13, 624–630. 10.1038/nmat3944. 24747780

[ref32] Bonaccorso F.; Bartolotta A.; Coleman J. N.; Backes C. 2D-Crystal-Based Functional Inks. Adv. Mater. 2016, 28, 6136–6166. 10.1002/adma.201506410. 27273554

[ref33] Kang J.; Sangwan V. K.; Wood J. D.; Hersam M. C. Solution-Based Processing of Monodisperse Two-Dimensional Nanomaterials. Acc. Chem. Res. 2017, 50, 943–951. 10.1021/acs.accounts.6b00643. 28240855

[ref34] Hu G. H.; Kang J.; Ng L. W. T.; Zhu X. X.; Howe R. C. T.; Jones C. G.; Hersam M. C.; Hasan T. Functional inks and printing of two-dimensional materials. Chem. Soc. Re.v 2018, 47, 3265–3300. 10.1039/C8CS00084K. 29667676

[ref35] Lin Z. Y.; Huang Y.; Duan X. F. Van der Waals thin-film electronics. Nat. Electron. 2019, 2, 378–388. 10.1038/s41928-019-0301-7.

[ref36] Lin Z. Y.; Liu Y.; Halim U.; Ding M. N.; Liu Y. Y.; Wang Y. L.; Jia C. C.; Chen P.; Duan X. D.; Wang C.; Song F.; Li M. F.; Wan C. Z.; Huang Y.; Duan X. F. Solution-processable 2D semiconductors for high-performance large-area electronics. Nature 2018, 562, 254 10.1038/s41586-018-0574-4. 30283139

[ref37] Jariwala D.; Sangwan V. K.; Lauhon L. J.; Marks T. J.; Hersam M. C. Carbon nanomaterials for electronics, optoelectronics, photovoltaics, and sensing. Chem. Soc. Rev. 2013, 42, 2824–2860. 10.1039/C2CS35335K. 23124307

[ref38] Eda G.; Yamaguchi H.; Voiry D.; Fujita T.; Chen M. W.; Chhowalla M. Photoluminescence from Chemically Exfoliated MoS2. Nano Lett. 2011, 11, 5111–5116. 10.1021/nl201874w. 22035145

[ref39] Jeong S.; Yoo D.; Ahn M.; Miro P.; Heine T.; Cheon J. Tandem intercalation strategy for single-layer nanosheets as an effective alternative to conventional exfoliation processes. Nat. Commun. 2015, 6, 6763 10.1038/ncomms6763. 25575047

[ref40] Abdelkader A. M.; Cooper A. J.; Dryfe R. A. W.; Kinloch I. A. How to get between the sheets: a review of recent works on the electrochemical exfoliation of graphene materials from bulk graphite. Nanoscale 2015, 7, 6944–6956. 10.1039/C4NR06942K. 25703415

[ref41] Park S.; Ruoff R. S. Chemical methods for the production of graphenes. Nat. Nanotechnol. 2009, 4, 217–224. 10.1038/nnano.2009.58. 19350030

[ref42] Kim M.; Seo J.; Kim J.; Moon J. S.; Lee J.; Kim J. H.; Kang J.; Park H. High-Crystalline Monolayer Transition Metal Dichalcogenides Films for Wafer-Scale Electronics. ACS Nano 2021, 15, 3038–3046. 10.1021/acsnano.0c09430. 33512141

[ref43] Kim J.; Kim S.; Cho Y. S.; Choi M.; Jung S. H.; Cho J. H.; Whang D.; Kang J. Solution-Processed MoS2 Film with Functional Interfaces via Precursor-Assisted Chemical Welding. ACS Appl. Mater. Inter. 2021, 13, 12221–12229. 10.1021/acsami.1c00159. 33657809

[ref44] Niyogi S.; Bekyarova E.; Itkis M. E.; McWilliams J. L.; Hamon M. A.; Haddon R. C. Solution properties of graphite and graphene. J. Am. Chem. Soc. 2006, 128, 7720–7721. 10.1021/ja060680r. 16771469

[ref45] Kang J.; Sangwan V. K.; Wood J. D.; Liu X.; Balla I.; Lam D.; Hersam M. C. Layer-by-Layer Sorting of Rhenium Disulfide via High-Density Isopycnic Density Gradient Ultracentrifugation. Nano Lett. 2016, 16, 7216–7223. 10.1021/acs.nanolett.6b03584. 27700101

[ref46] Kang J.; Seo J. W. T.; Alducin D.; Ponce A.; Yacaman M. J.; Hersam M. C. Thickness sorting of two-dimensional transition metal dichalcogenides via copolymer-assisted density gradient ultracentrifugation. Nat. Commun. 2014, 5, 5478 10.1038/ncomms6478. 25391315

[ref47] Fan X. B.; Xu P. T.; Zhou D. K.; Sun Y. F.; Li Y. G. C.; Nguyen M. A. T.; Terrones M.; Mallouk T. E. Fast and Efficient Preparation of Exfoliated 2H MoS2 Nanosheets by Sonication-Assisted Lithium Intercalation and Infrared Laser-Induced 1T to 2H Phase Reversion. Nano Lett. 2015, 15, 5956–5960. 10.1021/acs.nanolett.5b02091. 26288218

[ref48] Cho Y. S.; Rhee D.; Kim H. J.; Kim H. S.; Baik J. M.; Kang J. Solution-Processed Graphene Thin-Film Enables Binder-Free, Efficient Loading of Nanocatalysts for Electrochemical Water Splitting. Adv. Mater. Inter. 2021, 8, 2101576 10.1002/admi.202101576.

[ref49] Lin Z. Y.; Chen Y.; Yin A. D.; He Q. Y.; Huang X. Q.; Xu Y. X.; Liu Y. A.; Zhong X.; Huang Y.; Duan X. F. Solution Processable Colloidal Nanoplates as Building Blocks for High-Performance Electronic Thin Films on Flexible Substrates. Nano Lett. 2014, 14, 6547–6553. 10.1021/nl503140c. 25343683

[ref50] Yan Z.; Xu D.; Lin Z.; Wang P.; Cao B.; Ren H.; Song F.; Wan C.; Wang L.; Zhou J.; Zhao X.; Chen J.; Huang Y.; Duan X. Highly stretchable van der Waals thin films for adaptable and breathable electronic membranes. Science 2022, 375, 852–859. 10.1126/science.abl8941. 35201882

[ref51] Kang J.; Wells S. A.; Wood J. D.; Lee J. H.; Liu X. L.; Ryder C. R.; Zhu J.; Guest J. R.; Husko C. A.; Hersam M. C. Stable aqueous dispersions of optically and electronically active phosphorene. Proc. Natl. Acad. Sci. U.S.A. 2016, 113, 11688–11693. 10.1073/pnas.1602215113. 27092006 PMC5081619

[ref52] Kang J.; Wood J. D.; Wells S. A.; Lee J. H.; Liu X. L.; Chen K. S.; Hersam M. C. Solvent Exfoliation of Electronic-Grade, Two-Dimensional Black Phosphorus. ACS Nano 2015, 9, 3596–3604. 10.1021/acsnano.5b01143. 25785299

[ref53] Kang J.; Sangwan V. K.; Lee H. S.; Liu X. L.; Hersam M. C. Solution-Processed Layered Gallium Telluride Thin-Film Photodetectors. ACS Photonics 2018, 5, 3996–4002. 10.1021/acsphotonics.8b01066.

[ref54] Kang J.; Wells S. A.; Sangwan V. K.; Lam D.; Liu X.; Luxa J.; Sofer Z.; Hersam M. C. Solution-Based Processing of Optoelectronically Active Indium Selenide. Adv. Mater. 2018, 30, 1802990 10.1002/adma.201802990. 30095182

[ref55] Sangwan V. K.; Rangnekar S. V.; Kang J.; Shen J.; Lee H.-S.; Lam D.; Shen J.; Liu X.; Moraes A. C. M.; Kuo L.; Gu J.; Wang H.; Hersam M. C. Visualizing Thermally Activated Memristive Switching in Percolating Networks of Solution-Processed 2D Semiconductors. Adv. Funct. Mater. 2021, 31, 2107385 10.1002/adfm.202107385.

[ref56] Gao X. X.; Yin J.; Bian G.; Liu H. Y.; Wang C. P.; Pang X. X.; Zhu J. High-mobility patternable MoS2 percolating nanofilms. Nano Res. 2021, 14, 2255–2263. 10.1007/s12274-020-3218-6.

[ref57] Kim J.; Rhee D.; Song O.; Kim M.; Kwon Y. H.; Lim D. U.; Kim I. S.; Mazanek V.; Valdman L.; Sofer Z.; Cho J. H.; Kang J. All-Solution-Processed Van der Waals Heterostructures for Wafer-Scale Electronics. Adv. Mater. 2022, 34, 2106110 10.1002/adma.202106110. 34933395

[ref58] Jo S. B.; Kang J.; Cho J. H. Recent Advances on Multivalued Logic Gates: A Materials Perspective. Adv. Sci. 2021, 8, 2004216 10.1002/advs.202004216. PMC8061388 33898193

[ref59] Kim J.; Jung M.; Lim D. U.; Rhee D.; Jung S. H.; Cho H. K.; Kim H. K.; Cho J. H.; Kang J. Area-Selective Chemical Doping on Solution-Processed MoS2 Thin-Film for Multi-Valued Logic Gates. Nano Lett. 2022, 22, 570–577. 10.1021/acs.nanolett.1c02947. 34779637

[ref60] Suk J. W.; Kitt A.; Magnuson C. W.; Hao Y. F.; Ahmed S.; An J. H.; Swan A. K.; Goldberg B. B.; Ruoff R. S. Transfer of CVD-Grown Monolayer Graphene onto Arbitrary Substrates. ACS Nano 2011, 5, 6916–6924. 10.1021/nn201207c. 21894965

[ref61] Lin J. H.; Lin Y. C.; Wang X. S.; Xie L. M.; Suenaga K. Gentle transfer method for water-and acid/alkali-sensitive 2D materials for (S)TEM study. APL Mater. 2016, 4, 116108 10.1063/1.4967938.

[ref62] Kumar P.; Lynch J.; Song B. K.; Ling H. N.; Barrera F.; Kisslinger K.; Zhang H. Q.; Anantharaman S. B.; Digani J.; Zhu H. Y.; Choudhury T. H.; McAleese C.; Wang X. C.; Conran B. R.; Whear O.; Motala M. J.; Snure M.; Muratore C.; Redwing J. M.; Glavin N. R.; Stach E. A.; Davoyan A. R.; Jariwala D. Light-matter coupling in large-area van der Waals superlattices. Nat. Nanotechnol. 2022, 17, 182 10.1038/s41565-021-01023-x. 34857931

[ref63] Kelly A. G.; Hallam T.; Backes C.; Harvey A.; Esmaeily A. S.; Godwin I.; Coelho J.; Nicolosi V.; Lauth J.; Kulkarni A.; Kinge S.; Siebbeles L. D. A.; Duesberg G. S.; Coleman J. N. All-printed thin-film transistors from networks of liquid-exfoliated nanosheets. Science 2017, 356, 69–72. 10.1126/science.aal4062. 28386010

[ref64] McManus D.; Vranic S.; Withers F.; Sanchez-Romaguera V.; Macucci M.; Yang H. F.; Sorrentino R.; Parvez K.; Son S. K.; Iannaccone G.; Kostarelos K.; Fiori G.; Casiraghi C. Water-based and biocompatible 2D crystal inks for all-inkjet-printed heterostructures. Nat. Nanotechnol. 2017, 12, 343–350. 10.1038/nnano.2016.281. 28135260

[ref65] Zhang C. F.; Park S. H.; Ronan O.; Harvey A.; Seral-Ascaso A.; Lin Z. F.; McEvoy N.; Boland C. S.; Berner N. C.; Duesberg G. S.; Rozier P.; Coleman J. N.; Nicolosi V. Enabling Flexible Heterostructures for Li-Ion Battery Anodes Based on Nanotube and Liquid-Phase Exfoliated 2D Gallium Chalcogenide Nanosheet Colloidal Solutions. Small 2017, 13, 1701677 10.1002/smll.201701677. 28692755

[ref66] Zhang C. F.; McKeon L.; Kremer M. P.; Park S. H.; Ronan O.; Seral-Ascaso A.; Barwich S.; Coileain C. O.; McEvoy N.; Nerl H. C.; Anasori B.; Coleman J. N.; Gogotsi Y.; Nicolosi V. Additive-free MXene inks and direct printing of micro-supercapacitors. Nat. Commun. 2019, 10, 1795 10.1038/s41467-019-09398-1. 30996224 PMC6470171

[ref67] Amani M.; Lien D.-H.; Kiriya D.; Xiao J.; Azcatl A.; Noh J.; Madhvapathy S. R.; Addou R.; KC S.; Dubey M.; Cho K.; Wallace R. M.; Lee S.-C.; He J.-H.; Ager J. W.; Zhang X.; Yablonovitch E.; Javey A. Near-unity photoluminescence quantum yield in MoS2. Science 2015, 350, 1065–1068. 10.1126/science.aad2114. 26612948

